# Skin CO_2_ sniffing for wearable metabolic monitoring

**DOI:** 10.1126/sciadv.aec2376

**Published:** 2026-02-25

**Authors:** Seung-Rok Kim, Noelle Davis, Kalynna Tang, George A. Brooks, Ali Javey

**Affiliations:** ^1^ Department of Electrical Engineering and Computer Sciences, University of California, Berkeley, CA 94720, USA.; ^2^ Materials Sciences Division, Lawrence Berkeley National Laboratory, CA 94720, USA.; ^3^ Berkeley Sensor and Actuator Center, University of California, Berkeley, CA 94720, USA.; ^4^ Department of Bioengineering, University of California, Berkeley, CA 94720, USA.; ^5^ Department of Integrative Biology, University of California, Berkeley, CA 94720, USA.; ^6^ Kavli Energy NanoScience Institute, University of California, Berkeley, CA 94720, USA.

## Abstract

CO_2_ is a key physiological parameter used to assess hypoventilation and to estimate metabolic rates. However, current CO_2_ monitoring relies on bulky breath-analysis systems that are impractical for continuous use in daily life. Here, we present a wearable on-skin gas-sniffing system that quantifies skin-emitted CO_2_ and establishes its physiological relevance through direct correlation with exhaled CO_2_ and metabolic rate. Participant studies demonstrate strong correlations between CO_2_ flow rates from the skin and breath during both rest and exercise, with skin-emitted CO_2_ approximately four orders of magnitude lower than exhaled CO_2_. Skin-emitted CO_2_ also correlates with metabolic rate, suggesting its potential as a surrogate for breath-based indirect calorimetry. With its wearable form factor and physiological relevance, this skin gas sniffing system enables continuous, noninvasive metabolic monitoring and opens opportunities for studying skin gas exchange.

## Teaser

CO_2_ released from the skin offers an alternative to breath for tracking metabolism.

## INTRODUCTION

Metabolism is the chemical process of converting nutrients into energy to power vital bodily functions (*1*). Metabolic rate, a key indicator, shows how quickly the body consumes energy for its various processes. It varies substantially across activities for an individual, from minimal levels during sleep or fasting rest to much higher levels after eating or during exercise. When no external work is done, all the energy expended in the body eventually turns into heat. Therefore, direct calorimetry, which measures the body’s heat production, accurately estimates the basal or resting metabolic rate (*2*, *3*). However, this technique is impractical for physical activities due to challenges in capturing rapid heat changes and its restrictive technology (*4*–*6*).

Metabolic carts are widely adopted, using indirect calorimetry to track and analyze exhaled gases (*7*). On the basis of the principles of oxidative metabolism, measuring consumed O_2_ (VO_2_) and generated CO_2_ (VCO_2_) allows calculating both the respiratory quotient and the metabolic rate. Nevertheless, the requirement of a tightly sealed respiratory mask for accurate gas measurement presents considerable practical limitations (*8*). This setup restricts continuous monitoring during activities like eating (to assess diet-induced thermogenesis) or sleeping (for basal metabolic rate assessment), and it is often unsuitable for patients with pulmonary restrictions (*2*, *9*).

Consequently, there is a clear demand for more convenient, wearable devices that can be seamlessly integrated into daily activities for continuous metabolic assessment. Beyond breath analysis, the skin has been recognized as an alternative pathway for physiological monitoring. Humidity-based sensing has revealed that sweat-induced water vapor correlates with the amount of sweat secreted (*10*). More recently, wearable epidermal molecular flux platforms have demonstrated that transepidermal water loss, volatile organic compounds, and CO_2_ can be continuously monitored using wearable chamber systems on the skin (*11*). These findings highlight that the skin can serve as a physiologically informative interface, yet the direct quantitative link between skin-emitted gases and whole-body metabolism remains unexplored, particularly for CO_2_, a primary product of oxidative metabolism.

Here, we present the first attempt to correlate skin-emitted CO_2_ (skin CO_2_) with exhaled CO_2_ (breath CO_2_) and metabolic rate using a wearable on-skin CO_2_ sniffing system. The device uses a semi-open housing to stably detect the low flux of skin CO_2_, and finite element method (FEM) analysis converts steady-state chamber signals into quantitative skin CO_2_ flux. In participant studies, we simultaneously measure skin CO_2_, breath CO_2_, and metabolic rate during rest and constant-power exercise, enabling direct comparison. These findings show that skin CO_2_ reflects physiologically meaningful metabolic information. With its wearable form factor and quantitative sensing capability, this skin CO_2_ sniffing system provides a minimally obtrusive approach for continuous, noninvasive metabolic assessment.

## RESULTS

### Wearable skin CO_2_ sniffing

**Fig. 1.**
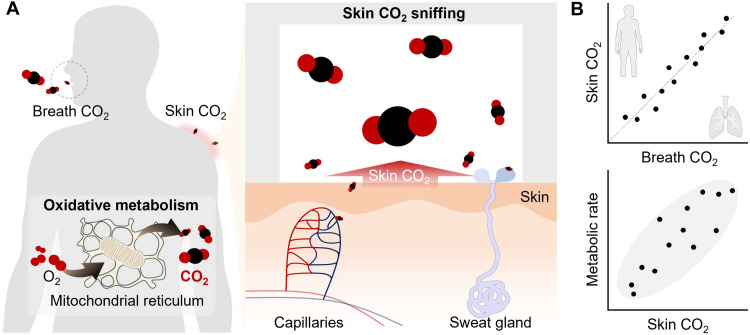
Concept of skin CO_2_ sniffing. (**A**) Schematic illustration showing that CO_2_ generated by oxidative metabolism is released not only through breath but also through the skin, where it can be captured by an on-skin chamber. (**B**) Conceptual relationships among skin CO_2_, breath CO_2_, and metabolic rate, highlighting the potential of skin CO_2_ as an indicator of whole-body metabolic activity.

As illustrated in Fig. 1A, CO_2_ is generated by oxidative metabolism within the mitochondrial reticulum (*12*), where cellular respiration consumes O_2_ and produces CO_2_ as part of energy production. Most CO_2_ enters the capillaries and is transported to the lungs for external respiration as breath CO_2_. A smaller fraction reaches the skin surface via transcutaneous diffusion or by dissolving into sweat and exiting through the sweat gland (*13*, *14*). This cutaneous pathway provides an opportunity to probe the physiological coupling between skin CO_2_ and whole-body metabolism.

Figure 1B conceptually summarizes the relationships among skin CO_2_, breath CO_2_, and metabolic rate. Although skin CO_2_ is orders of magnitude lower than breath CO_2_, participant studies show that the two maintain a consistent linear relationship, suggesting that skin CO_2_ can serve as a minimally obtrusive indicator of metabolic activity.

### Wearable skin CO_2_ sniffer

**Fig. 2.**
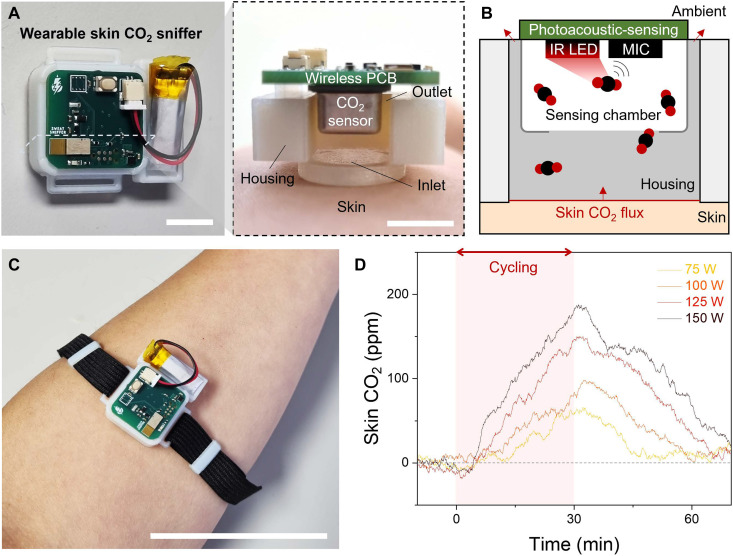
Wearable skin CO_2_ sniffer and device structure. (**A**) Photograph of the wearable skin CO_2_ sniffer and its corresponding cross-sectional view showing the internal geometry. Scale bars, 1 cm. (**B**) Schematic illustration of the photoacoustic CO_2_ sensing mechanism and the semi-open housing. (**C**) Photograph of the device attached to the forearm. Scale bar, 5 cm. (**D**) Representative skin CO_2_ time series measured during stationary cycling at different power outputs.

As shown in Fig. 2A, the wearable skin CO_2_ sniffer integrates a commercial photoacoustic CO_2_ sensor mounted on a custom wireless printed circuit board (PCB) and enclosed within a 3D-printed wearable housing (see Materials and Methods). Although typical skin CO_2_ flux is extremely low (approximately 100 nmol m^−2^ s^−1^) (*15*–*17*), sealing the housing against the skin creates a confined space that passively enriches the CO_2_ to levels detectable by the sensor. The housing also incorporates a venting outlet that continuously releases excess gas, preventing saturation and pressure buildup.

Figure 2B illustrates the sensing mechanism. Skin CO_2_ flux increases the local concentration within the confined chamber relative to ambient air. While excess gas is vented to maintain a steady state, the photoacoustic sensor operates using an onboard infrared (IR) light-emitting diode and microphone (MIC). IR excitation induces CO_2_ vibrational absorption, generating pressure waves that are subsequently detected by MIC.

Figure 2C shows the integrated device worn on the forearm. As a preliminary demonstration, the sniffer successfully tracked skin CO_2_ dynamics during stationary cycling at power outputs of 75, 100, 125, and 150 W (Fig. 2D). The skin CO_2_ traces were baseline-corrected by subtracting ambient CO_2_ and exhibited clear postexercise recovery toward resting levels.

### FEM simulation for characterization of the wearable skin CO_2_ sniffer

**Fig. 3.**
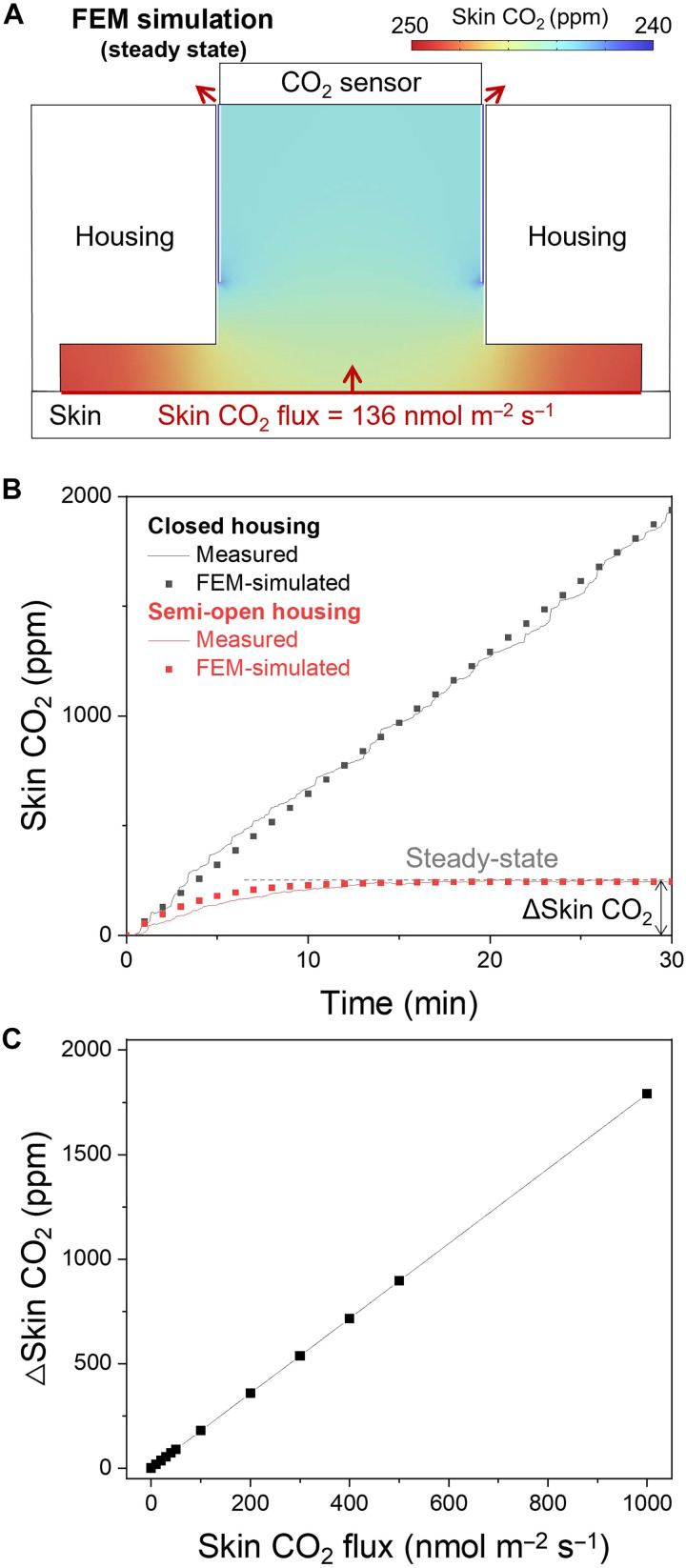
FEM simulation characterizing skin CO_2_ accumulation in the semi-open housing. (**A**) FEM-simulated cross-sectional skin CO_2_ distribution within the semi-open housing at steady state for a skin CO_2_ flux of 136 nmol m^−2^ s^−1^. (**B**) Comparison of measured and FEM-simulated skin CO_2_ transients for a closed housing (black) and a semi-open housing (red). ΔSkin CO_2_ is defined as the steady-state skin CO_2_ concentration relative to ambient CO_2_. (**C**) Linear relationship between the imposed skin CO_2_ flux and the resulting Δskin CO_2_ obtained from FEM simulations.

To quantitatively relate the accumulated skin CO_2_ to its underlying flux, we performed FEM simulations. Figure 3A shows the steady-state skin CO_2_ distribution within the semi-open housing, where the concentration stabilizes as the skin-driven influx is balanced by the outflux through the vent. To capture transient behavior and the role of venting, Fig. 3B compares closed and semi-open configurations. The closed housing exhibits a continuous rise in skin CO_2_ due to the absence of outward flow, whereas the semi-open design reaches a steady state. The measured transients agree with the simulated responses, yielding an estimated skin CO_2_ flux of 136 nmol m^−2^ s^−1^ from the closed-housing rise. We then defined Δskin CO_2_ as the steady-state chamber concentration relative to ambient and evaluated its dependence on flux. As shown in Fig. 3C, FEM simulations reveal a linear relationship between flux and Δskin CO_2_, enabling direct extraction of skin CO_2_ flux from the wearable sniffer.

### Simultaneous measurements of skin CO_2_ and breath CO_2_

**Fig. 4.**
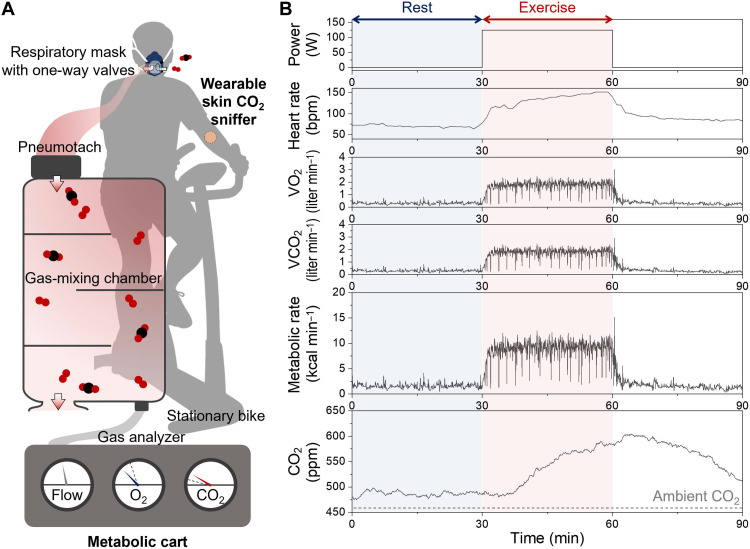
Qualitative comparison between the skin CO_2_ sniffer and the metabolic cart. (**A**) Schematic of a participant instrumented with a metabolic cart for real-time respiratory gas analysis and wearing a wearable skin CO_2_ sniffer on the forearm during stationary cycling. (**B**) Simultaneous time series data from both systems during rest and constant-power cycling. Blue and red regions indicate rest and exercise periods, respectively.

To evaluate how skin CO_2_ relates to whole-body metabolic activity, we acquired simultaneous measurements from the wearable sniffer and a metabolic cart. As shown in Fig. 4A, participants wore a respiratory mask connected to a clinical metabolic cart while the wearable skin CO_2_ sniffer was mounted on the forearm during constant-power stationary cycling (see Materials and Methods). The metabolic cart provided breath-by-breath VO_2_ and VCO_2_ measurements, from which metabolic rate was calculated using the Weir equation (*18*). Exhaled gases passed through a pneumotach for flow measurement, were mixed in a chamber, and subsequently sampled for analysis. While this system enables highly accurate respiratory gas quantification, the sealed mask and benchtop instrumentation make the setup substantially more obtrusive than the wearable sniffer.

As shown in Fig. 4B, each participant completed sequential resting (30 min) and exercising (30 min) periods, while breath gases, skin CO_2_, and heart rate were recorded simultaneously. During rest, VO_2_, VCO_2_, and metabolic rate exhibited stable steady-state values. Upon the transition to constant-power exercise, all breath-derived parameters increased and then stabilized at higher steady-state levels. Skin CO_2_ followed the same qualitative behavior, reaching a resting steady state and then rising during exercise before plateauing at a higher steady-state concentration.

### Participant study for quantitative correlation between skin CO_2_ and breath CO_2_

**Fig. 5.**
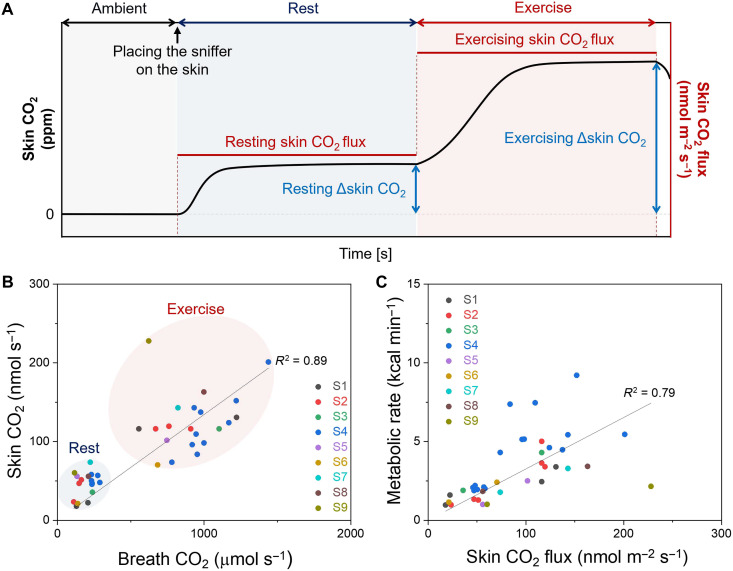
Quantitative metabolic assessment using skin CO_2_ during rest and exercise. (**A**) Schematic representation of the skin CO_2_ time series showing steady-state levels during rest and constant-power exercise. ΔSkin CO_2_ values at steady state are used to determine skin CO_2_ flux based on the FEM-derived calibration. (**B**) Correlation between skin CO_2_ emission rate and breath CO_2_ exhalation rate across nine participants during rest and exercise. (**C**) Correlation between metabolic rate measured by the metabolic cart and skin CO_2_ flux across nine participants under both conditions.

Using the FEM-derived calibration, we quantify skin CO_2_ flux during consecutive 30-min rest and exercise periods and directly compare these values with breath CO_2_. As illustrated in Fig. 5A, skin CO_2_ rises immediately after placing the sniffer on the skin and reaches a resting steady state. The resting Δskin CO_2_ is then converted into a resting skin CO_2_ flux. Following the transition to exercise, a new steady-state Δskin CO_2_ emerges, enabling calculation of the exercising skin CO_2_ flux. Because participants are simultaneously connected to a metabolic cart, breath CO_2_ values are recorded in parallel. For comparison on a whole-body basis, skin CO_2_ flux measured on the forearm is scaled using a representative body surface area of 2 m^2^.

As shown in Fig. 5B, skin CO_2_ flux strongly correlates with breath CO_2_ exhalation rate across nine participants during both rest and exercise (*R*^2^ = 0.89), even though skin CO_2_ levels are three to four orders of magnitude smaller. Since breath CO_2_ is routinely used to estimate metabolic rate, we further evaluate whether skin CO_2_ reflects metabolic activity. Figure 5C shows that skin CO_2_ emission rate also correlates with metabolic rate (*R*^2^ = 0.79). While the present analysis relies on steady-state values, future studies may investigate transient responses and individual variability.

## DISCUSSION

In this study, we establish the first quantitative link between skin CO_2_, breath CO_2_, and metabolic rate. The semi-open chamber design enables reliable skin CO_2_ measurements by preventing gas accumulation, and FEM analysis provides a quantitative framework for converting steady-state chamber concentrations into skin CO_2_ flux. Participant studies reveal a strong positive correlation between skin CO_2_ and breath CO_2_, as well as a consistent positive correlation with metabolic rate. These findings demonstrate that skin CO_2_ carries physiologically meaningful metabolic information. We envision this skin gas sniffing approach being extended to studies of metabolic physiology and other skin-related gas pathways. Further improvements in active gas handling and sensor integration are expected to further improve accuracy and expand the range of physiological and clinical applications.

## MATERIALS AND METHODS

### Wireless PCB for CO_2_ sensor

A custom-designed wireless PCB was developed in-house to integrate the commercial photoacoustic CO_2_ sensor (SCD41, Sensirion AG, Switzerland). The SCD41 was selected for its high precision and photoacoustic detection mechanism, which is more sensitive than thermal conductivity–based CO_2_ sensors. The nominal measurement accuracy is ± [50 parts per million (ppm) + 5% of reading] within 400 to 5000 ppm, with higher precision at lower concentration range typically observed on-skin (500–600 ppm). The sensor also incorporates on-chip temperature and humidity compensation, minimizing potential artifacts arising from skin heat dissipation and sweating. To verify that environmental variations do not confound skin CO_2_ measurements, we experimentally test the sniffer under controlled temperature and humidity perturbations (fig. S1). In the sniffer, the SCD41 operated in high-performance mode with measurements taken every 5 s. Data were acquired over I^2^C protocol by a Nordic nRF52832 microcontroller, as shown in fig. S2, and wirelessly transmitted to iOS smartphone application via Bluetooth Low Energy.

### Fabrication of semi-open housing

The housings were fabricated using an Objet260 3D printer. Rigid components defining the gas pathway were printed using a digital ABS blend (RGD531 and RGD515), while the soft skin-contacting inlet was printed using Agilus30 Clear. VHB double-sided tape (3M) was used to seal for the closed-housing configuration and to define the outlet geometry for the semi-open housing. A more detailed description of the 3D printing process was included in a previous study (*10*).

### Gas dynamics analysis through FEM simulation

FEM simulations were performed using the Transport of Diluted Species module in COMSOL Multiphysics (COMSOL Inc., USA). CO_2_ was modeled as a diluted species in the air with an isotropic diffusion coefficient of 1.6 × 10^−5^ m^2^ s^−1^. A full 3D geometry corresponding to the actual housing was used. The skin boundary was assigned to a flux condition spanning physiologically relevant skin CO_2_ flux levels. The total amount of CO_2_ in the sensing chamber was obtained by volumetric integration over the chamber volume (8 mm × 8 mm × 5.5 mm). To match the experimental definition of skin CO_2_ (ambient-subtracted CO_2_), we set ambient CO_2_ to zero in the FEM simulations. This approach is mathematically equivalent to subtracting ambient concentration in postprocessing.

### On-body participant study

On-body human trials were carried out at the University of California, Berkeley in compliance with the human research protocol (CPHS 2014-08-6636) approved by the Berkeley Institutional Review Board. Informed consent was obtained from the participants before enrolling in the study.

To obtain simultaneous skin CO_2_ and breath CO_2_, nine participants wore a respiratory mask connected to a metabolic cart along with the wearable skin CO_2_ sniffer on the forearm. A commercial activity tracker (Fitbit) was used to monitor heart rate. Before each experiment, participants rested for 10 to 30 min to ensure physiological stabilization. After the sensor placement, participants rested for an additional 30 min to establish baseline skin CO_2_ values.

All trials were conducted during the daytime in a controlled laboratory environment maintained at 20° to 25°C and 40 to 60% relative humidity. Ambient CO_2_ levels were measured before each trial and remained stable throughout the experiments. Participants then completed 30 min of stationary cycling at power levels between 50 and 150 W. When multiple trials were performed, they were conducted on different days to ensure consistency and comfort. Exercise intensity was increased only when the participant felt comfortable. After each exercise session, participants rested for 10 to 60 min. No data points were excluded except for occasional sensor dropouts predefined in the analysis protocol.
