# Supplementary material for: Skin CO2 sniffing for wearable metabolic monitoring
Source: Sci Adv. 2026 Feb 25;12(9):eaec2376. doi: 10.1126/sciadv.aec2376 (PMC12935033; doi:10.1126/sciadv.aec2376)
Supplement: Supplementary file 1 — Figs. S1 and S2 [file sciadv.aec2376_sm.pdf]

Science Advances
AAAS

Supplementary Materials for

Skin CO_2 sniffing for wearable metabolic monitoring

Seung-Rok Kim *et al.*

Corresponding author: Ali Javey, ajavey@berkeley.edu

Sci. Adv. **12**, eaec2376 (2026)
DOI: 10.1126/sciadv.aec2376

This PDF file includes:

Figs. S1 and S2

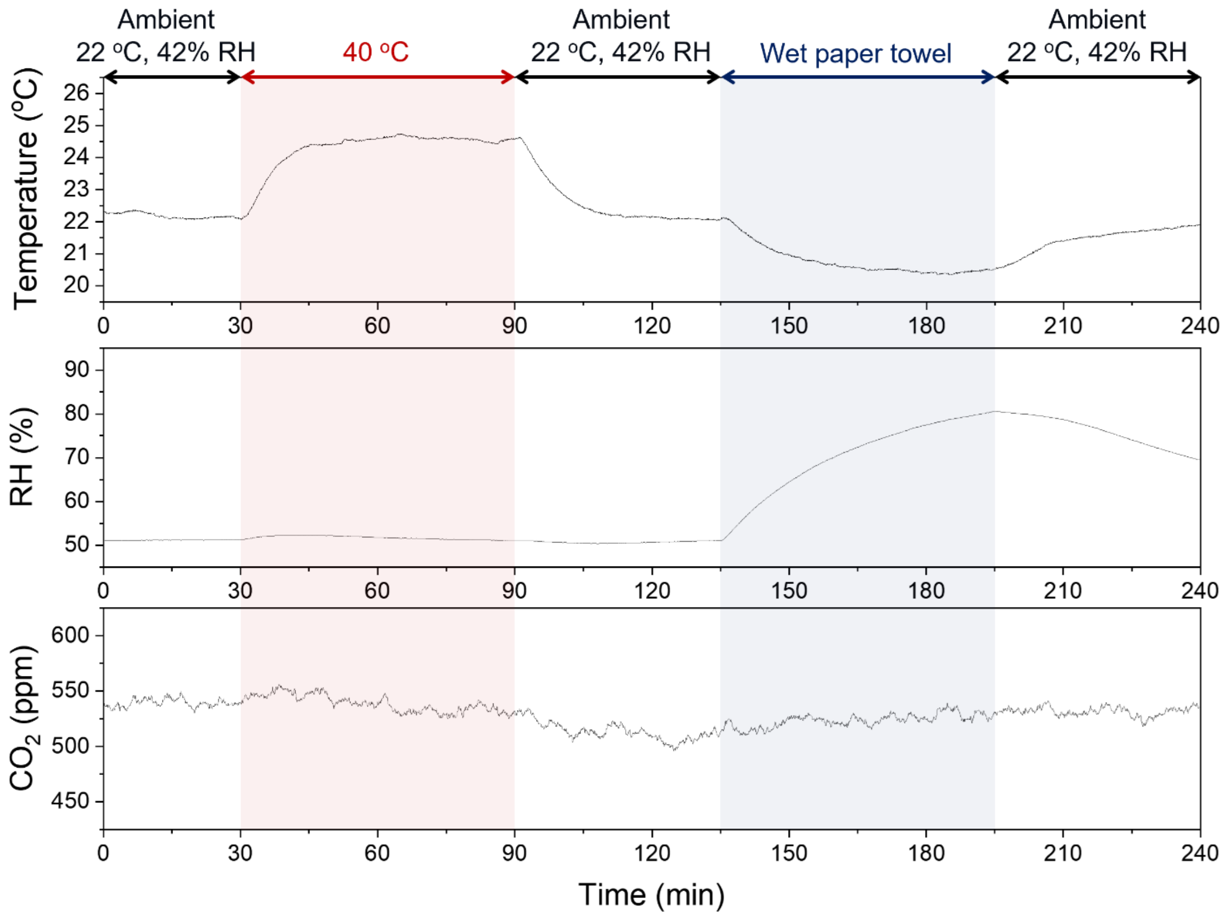

Fig. S1.

Continuous CO_2 measurement under controlled temperature and humidity variations.

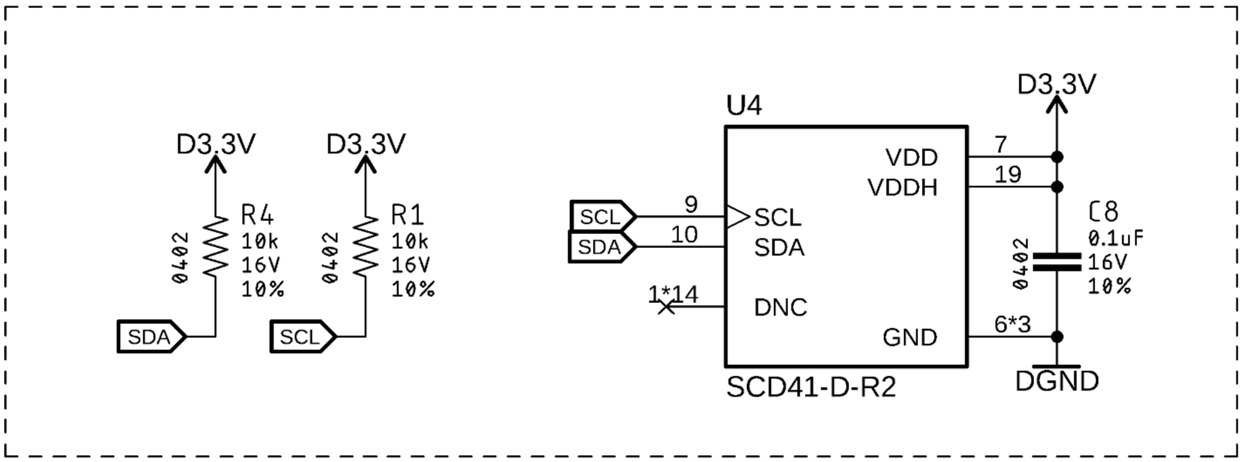

Fig. S2.

Circuit schematic for I^2C communication with CO_2 sensor (Sensirion SCD41).
